# Measuring group‑ and individual‑level change of FACIT- fatigue, NFLymSI-18 and PROMIS fatigue measures (4a, 7a and 13a) in lymphoma

**DOI:** 10.1186/s41687-026-01154-6

**Published:** 2026-07-19

**Authors:** Maja Kuharic, Kimberly A. Webster, Anne R. Zola, Maria E. Corona, Elijah A. Patten, Reem Karmali, Leo Gordon, Shuo Ma, John D. Peipert, David Cella

**Affiliations:** 1 Department of Medical Social Sciences, Northwestern University, Chicago, USA; 2 Robert H. Lurie Comprehensive Cancer Center, Northwestern University, Chicago, USA; 3 Centre for Patient Reported Outcomes Research, University of Birmingham, Birmingham, UK

**Keywords:** Fatigue, Lymphoma, Patient-reported outcomes, PROMIS, FACIT-fatigue, Responsiveness, Individual-level change, Reliable change index, Likely change index

## Abstract

**Background:**

Fatigue significantly impacts quality of life in lymphoma patients. Multiple patient-reported fatigue measures are used in lymphoma research, including FACIT-Fatigue and PROMIS Fatigue instruments, but direct comparisons of their responsiveness and ability to detect individual-level change in this population are lacking. This study evaluated the responsiveness and sensitivity to individual-level change of Functional Assessment of Chronic Illness Therapy-Fatigue (FACIT-Fatigue), Patient-Reported Outcomes Measurement Information System (PROMIS) Fatigue measures (4a, 7a, and 13a), and NFLyMSI-18 Lymphoma Symptom Index in patients with lymphoma.

**Methodology:**

In a prospective observational study, 102 lymphoma patients at an academic cancer center completed FACIT‑Fatigue, PROMIS Fatigue 4a, 7a, and 13a, and NFLymSI‑18 at baseline and again at 3 months (*n* = 89). Responsiveness was evaluated using standardized response means (SRMs) for patients classified as improved or worsened via clinical anchors: Patient Global Rating of Change, Patient Global Rating of Severity, Patient Global Rating of Severity Interference, and PRO-CTCAE severity and interference fatigue items. Individual-level change was evaluated using Reliable Change Index (RCI) and Likely Change Index (LCI).

**Results:**

SRM magnitudes varied by anchor and direction of change. For improvement, FACIT-Fatigue and NFLymSI-18 showed consistent responsiveness (SRMs 0.67–1.01 and 0.62–0.89, respectively), while PROMIS measures showed more variability (SRMs 0.31–1.58). For worsening, PROMIS measures, particularly 4a and 13a, demonstrated larger SRMs (0.75–1.71) than FACIT-Fatigue (0.73–1.24) or NFLymSI-18 (0.57–0.84) for most anchors. LCI analyses identified reliable improvement in 31.5% of patients using FACIT-Fatigue and 24.7–33.7% with PROMIS, and reliable worsening in 24.7% using FACIT-Fatigue and 23.6–34.8% using PROMIS measures.

**Conclusions:**

Among lymphoma patients, FACIT-Fatigue and PROMIS Fatigue measures are responsive to meaningful change, with somewhat differing magnitude estimates across anchors and direction of change. These findings support planning for the use and interpretation of these fatigue measures in future clinical trials.

**Supplementary Information:**

The online version contains supplementary material available at https://doi.org/10.1186/s41687-026-01154-6.

## Abstract (plain language summary)

Fatigue is one of the most common and troubling symptoms for people with lymphoma. It affects daily life, work, and recovery. To study and treat fatigue, researchers need good measures that can show when patients are feeling better or worse. We tested three fatigue questionnaires: FACIT-Fatigue, PROMIS Fatigue (with 4, 7, or 13 questions), and the Lymphoma Symptom Index (NFLymSI-18). Over 100 patients with lymphoma completed these at the start of the study and again three months later. All the questionnaires detected meaningful changes in fatigue, but the amount of change they showed depended on which tool was used and whether patients improved or worsened. Our findings can guide doctors and researchers in choosing the best fatigue measure for future studies.

**Supplementary Information:**

The online version contains supplementary material available at https://doi.org/10.1186/s41687-026-01154-6.

## Background

Fatigue is among the most prevalent and distressing symptoms experienced by patients with lymphoma, both during and after treatment [1, 2]. The Lymphoma Coalition’s 2018 Global Patient Survey found that 72% reported experiencing life-impacting fatigue [3]. Among long‑term survivors of non‑Hodgkin lymphoma, approximately one‑third report persistent, severe fatigue years after treatment [4]. Fatigue affects functional status, quality of life, ability to work and everyday activities [5]. 

Patient-reported outcomes (PROs) have gained increasing importance in oncology clinical trials and regulatory decisions [6]. The Functional Assessment of Chronic Illness Therapy-Fatigue (FACIT-Fatigue) and the Patient-Reported Outcomes Measurement Information System (PROMIS) Fatigue measures are widely used in cancer research and clinical settings [7]. The FACIT-Fatigue was initially developed for use in cancer settings, while PROMIS Fatigue measures were created using modern psychometric methods for use across various health conditions [8, 9]. The National Comprehensive Cancer Network-Functional Assessment of Cancer Therapy (NCCN-FACT) Lymphoma Symptom Index (NFLymSI-18) is an 18-item measure specifically developed for targeted assessment of key symptoms and concerns in lymphoma patients, including fatigue, pain, and emotional distress [10]. Both FACIT and PROMIS measurement systems have demonstrated strong psychometric properties, including reliability, validity, and sensitivity to change in diverse patient populations with cancer [11–13]. 

For PRO measures to effectively capture treatment effects and changes in disease symptoms, they must be responsive to clinically meaningful changes in the patient’s condition [14]. Responsiveness, defined as a “measure’s ability to detect change when true change has occurred,” is an important property that can vary by measurement instrument, patient population, and clinical context [15]. It is particularly important in clinical trials of novel therapeutics where detecting meaningful improvement or deterioration in patient-reported symptoms can influence regulatory and clinical decision-making [15]. Additionally, understanding important difference values for these measures helps interpret score changes and establish meaningful thresholds for responder definitions in clinical trials for a specific population [16]. In lymphoma specifically, where fatigue is both a disease symptom and treatment side effect, optimal measurement is important for assessment of treatment benefit [17]. The purpose of this study was to evaluate the responsiveness and sensitivity to individual-level changes of the FACIT-Fatigue, PROMIS Fatigue measures (4a, 7a, and 13a), and the NFLyMSI-18 in patients with lymphoma. We specifically aimed to: (1) determine which measures demonstrated the greatest sensitivity to change in this cohort, and (2) examine whether responsiveness differed based on the direction of change (improvement versus worsening).

## Methods

### Study design and participants

Participants with a histologically confirmed diagnosis of lymphoma were recruited from the Northwestern University Robert H. Lurie Comprehensive Cancer Center between September 2018 and February 2020. Eligible patients were ≥ 18 years old, about to start or currently receiving treatment, with a self-reported Eastern Cooperative Oncology Group (ECOG) performance status of ≤ 3, and the ability to understand and complete study questionnaires in English.

The study consisted of two participant groups: Group A included 24 patients enrolled prior to starting their next line of therapy; they completed assessments at baseline, seven days later (for a separate test-retest reliability analysis not the focus of this manuscript), and at three months. Group B included 78 patients at any point in their treatment trajectory who completed assessments at baseline and three months. All responsiveness and individual-level change analyses reported in this manuscript are based on changes from baseline to the three-month assessment for both groups. The study protocol was approved by the Institutional Review Board (STUD00206739), and all patients provided written informed consent.

### Procedures and measures

Eligible patients were recruited during clinical visits. Following consent, participants completed electronic questionnaires via Research Electronic Data Capture (REDCap) on a tablet (iPad) at baseline. Follow-up assessments were completed either remotely via email link or in-clinic using the same electronic platform. Participants received $50 for completing the baseline assessment and an additional $75 after completing the three-month assessment.

The assessment battery included the following measures:

#### FACIT-fatigue

A 13-item scale assessing fatigue and its impact on daily activities and function during the past 7 days. Items are rated on a 5-point scale (0 = Not at All to 4 = Very Much). Total scores range from 0 to 52, with higher scores indicating less fatigue [18]. 

#### NFLymSI‑18

An 18-item measure specific to lymphoma symptoms and concerns, including but not limited to fatigue. Items are rated on a 5-point scale (0 = Not at All to 4 = Very Much) with a 7-day recall period. Subscale scores are summed, with higher scores indicating better outcomes (less symptom burden) [10]. 

PROMIS Fatigue short forms included Items are rated on a 5-point scale (1 = Never/Not at All to 5 = Always/Very Much) with a 7-day recall period. **PROMIS Fatigue 7a** (a 7-item measure of fatigue experience and impact) and **PROMIS Fatigue 4a**: A 4-item brief measure of fatigue [19]. **PROMIS Fatigue 13a** content is identical to FACIT-Fatigue but with IRT-based scoring instead of simple summation scoring. All PROMIS measures were scored on the PROMIS T-score metric (mean = 50, SD = 10, referenced to a general population), with higher scores indicating greater fatigue.

The following anchors were collected to evaluate responsiveness [15]:

1. **Patient Global Rating of Change (PGRC)**: A 7-point scale directly assessing perceived change in fatigue since the previous assessment, ranging from “very much worse” to “very much better”.
2. **Patient Global Rating of Severity (PGRS1)**: A 5-point scale rating fatigue severity at each time point, from “none” to “very severe”.
3. **Patient Global Rating of Interference (PGRS2)**: A 5-point scale rating fatigue interference with daily life at each time point, from “not at all” to “extremely”.
4. **Patient-Reported Outcomes of the Common Terminology Criteria for Adverse Events (PRO-CTCAE) Fatigue items** [20, 21]: Patient-reported versions of the Common Terminology Criteria for Adverse Events (CTCAE) for fatigue severity (PRO-CTCAE Fatigue Severity) and interference (PRO-CTCAE Fatigue Interference). Items are scored individually on a 5-point scale (0 = None, 4 = Very severe or Very much), with a higher score indicating greater symptom severity or interference.

### Statistical analysis

Groups A and B were combined and treated as a single cohort. Responsiveness was evaluated using anchor-based methods. Sensitivity to individual-level change was assessed using distribution-based methods.

For anchor-based responsiveness analyses, patients were categorized as “improved,” “worsened,” or “stable” based on changes in the five anchors. For the PGRC anchor, patients reporting their fatigue as “a little better,” “moderately better,” or “very much better” were classified as “improved,” while those reporting “a little worse,” “moderately worse,” or “very much worse” were classified as “worsened.” For PGRS1 and PGRS2 anchors as well as PRO-CTCAE Fatigue Severity and Interference items, improvement or worsening was defined as at least a one-level change from baseline to follow-up. Standardized Response Means (SRMs) were calculated for each fatigue measure within improvement and worsening groups for each anchor. SRMs were computed as the mean change score divided by the standard deviation of that change, with values of 0.2, 0.5, and 0.8 considered small, medium, and large effect sizes, respectively. Anchor response categories were pooled into “improved” and “worsened” groups to ensure adequate sample sizes for stable SRM estimation. Unpooled SRM estimates by individual PGRC response level are provided in Supplementary Table S2 [14, 22]. We calculated Spearman correlation coefficients between change scores for each fatigue measure and the five anchor variables and examined responsiveness only among those with a correlation of *≥* 0.30 [15]. While initially collected, the daily version of PROMIS Fatigue (7b) was excluded from the main analysis due to inadequate correlations with clinical anchors (all correlation coefficients < 0.30). All other fatigue measures demonstrated sufficient correlations (coefficients ≥ 0.3) with all anchors (Table S1) [15]. 

To estimate statistically significant individual change thresholds, we calculated the Reliable Change Index (RCI) and Likely Change Index (LCI) [24, 25]. The RCI identifies score changes that exceed what would be expected by measurement error alone by evaluating whether an individual change score is statistically significantly greater than a difference that could have occurred due to random measurement error [24]. The LCI, a derivation of the RCI, helps determine whether an observed change score is sufficiently large enough to be attributable to factors beyond measurement error or chance. RCI uses a 95% confidence threshold, while LCI uses a 68% confidence threshold [23]. The RCI was calculated as: RCI = (X2 – X1) / Sdiff, where Sdiff = √2 × SEM, and SEM = SDbaseline × √(1 – α), with α being Cronbach’s coefficient alpha at baseline [24]. An RCI exceeding ± 1.96 indicates statistically significant change at a p-value of < 0.05. The LCI applies a threshold of ± 0.994 (approximately 68% confidence, equivalent to p-value of < 0.32) to identify changes more likely than not to reflect true change [23]. We calculated the frequency and proportion of patients classified as statistically significantly improved, worsened, or unchanged using both thresholds [25]. All analyses were conducted using SAS version 9.4 (SAS Institute, Cary, NC).

## Results

### Participant characteristics

A total of 102 patients with lymphoma were enrolled in the study, with 89 (87.3%) completing the 3-month follow-up assessment. Analyses were conducted on this subset of patients with complete baseline and follow-up data. The sample had a mean age of 61.0 years (SD = 14.9), and 48 (53.9%) were male. Most participants identified as non-Hispanic/Latino 74 (83.1%) and White 78 (87.6%). Lymphoma subtypes included B-cell NHL, T-cell NHL, and classical Hodgkin lymphoma, with B-cell histology representing the majority. At the time of enrollment, participants were about to start or were undergoing immunotherapy, chemoimmunotherapy, chemotherapy, or other targeted therapies; immunotherapy-containing regimens constituted the majority. Common self-reported comorbidities included cancer (other than lymphoma or non-melanoma skin cancer) 25 (28.1%), arthritis 24 (27.0%), anxiety 18 (20.2%), and diabetes 16 (18.0%). Table 1 presents the demographic and clinical characteristics of the analysis sample.

**Table 1:** Participants characteristics (*n* = 89)

Characteristic	*n* (%)
**Age**	
Mean (SD)	61.0 (14.9)
Median (range)	65 (21–85)
**Gender**	
Male	48 (53.9%)
Female	41 (46.1%)
**Ethnicity**	
Not Hispanic/Latino	74 (83.1%)
Hispanic/Latino	14 (15.7%)
Missing	1 (1.1%)
**Race**	
White	78 (87.6%)
Black or African American	4 (4.5%)
Asian	0 (0.0%)
Other	2 (2.2%)
American Indian or Alaskan Native	0 (0.0%)
Native Hawaiian or other Pacific Islander	4 (4.5%)
**Patient-rated ECOG Performance Status**	
0 – No symptoms	31 (34.8%)
1 - Some symptoms, no bedrest	25 (28.1%)
2 - Bedrest, but < 50% of waking day	24 (27.0%)
3 - Bedrest, but *≥* 50% of waking day	9 (10.1%)
**Comorbidities**	
Cancer (other than lymphoma or non-melanoma skin cancer)	25 (28.1%)
Arthritis, rheumatism, or lupus erythematosus	24 (27.0%)
Anxiety	18 (20.2%)
Diabetes or high blood sugar	16 (18.0%)
Asthma	15 (16.9%)
Migraines or severe headaches	13 (14.6%)
Depression	11 (12.4%)
Stroke, brain hemorrhage, or TIA	7 ( 7.9%)
Coronary artery disease	7 ( 7.9%)
Chronic lung disease (COPD), or chronic bronchitis or emphysema	5 ( 5.6%)
Angina or chest pain	4 ( 4.5%)
Heart failure or congestive failure	3 ( 3.4%)
Renal (kidney) disease	4 ( 4.5%)
Ulcers (stomach, duodenal, or peptic)	4 ( 4.5%)
Alcohol or drug problem	3 ( 3.4%)
Liver disease, hepatitis, or cirrhosis	2 ( 2.2%)
HIV or AIDS	1 ( 1.1%)
**Baseline Fatigue Scores**,** mean (SD)**	
FACIT-Fatigue	34.7 (9.4)
PROMIS Fatigue 4a T-score	51.3 (10.8)
PROMIS Fatigue 7a T-score	51.4 (9.9)
PROMIS Fatigue 13a T-score	51.8 (10.4)
NFLymSI-18 DRS-P	21.2 (5.3)
NFLymSI-18 DRS-E	10.6 (4.3)
NFLymSI-18 TSE	9.6 (2.9)
NFLymSI-18 FWB	10.2 (3.8)

Note: ECOG = Eastern Cooperative Oncology Group; PSR = Performance Status Rating. NFLymSI-18 subscales: DRS-P = Disease-Related Symptoms-Physical; DRS-E = Disease-Related Symptoms-Emotional; TSE = Treatment Side Effects; FWB = Function/Well-Being. Higher scores indicate better outcomes (less symptom burden)

### Responsiveness by anchor

#### Patient global rating of change (PGRC)

Among patients reporting improvement in fatigue experience, the PROMIS Fatigue 7a showed the largest SRM (0.72; mean change − 4.3), followed by FACIT-Fatigue (0.67; mean change + 5.0), PROMIS Fatigue 13a (0.52; mean change − 3.4), PROMIS Fatigue 4a (0.31; mean change − 2.8), and NFLymSI-18 (0.62; mean change + 4.4) (Fig. 1a). For patients reporting worsening in fatigue experience, PROMIS Fatigue 7a again showed the largest magnitude of response (SRM = 0.91; mean change + 6.3), followed by PROMIS Fatigue 13a (0.84; mean change + 5.8), PROMIS Fatigue 4a (0.75; mean change + 6.4), FACIT-Fatigue (0.73; mean change − 6.8), and NFLymSI-18 (0.64; mean change − 5.7).

**Fig. 1a:**
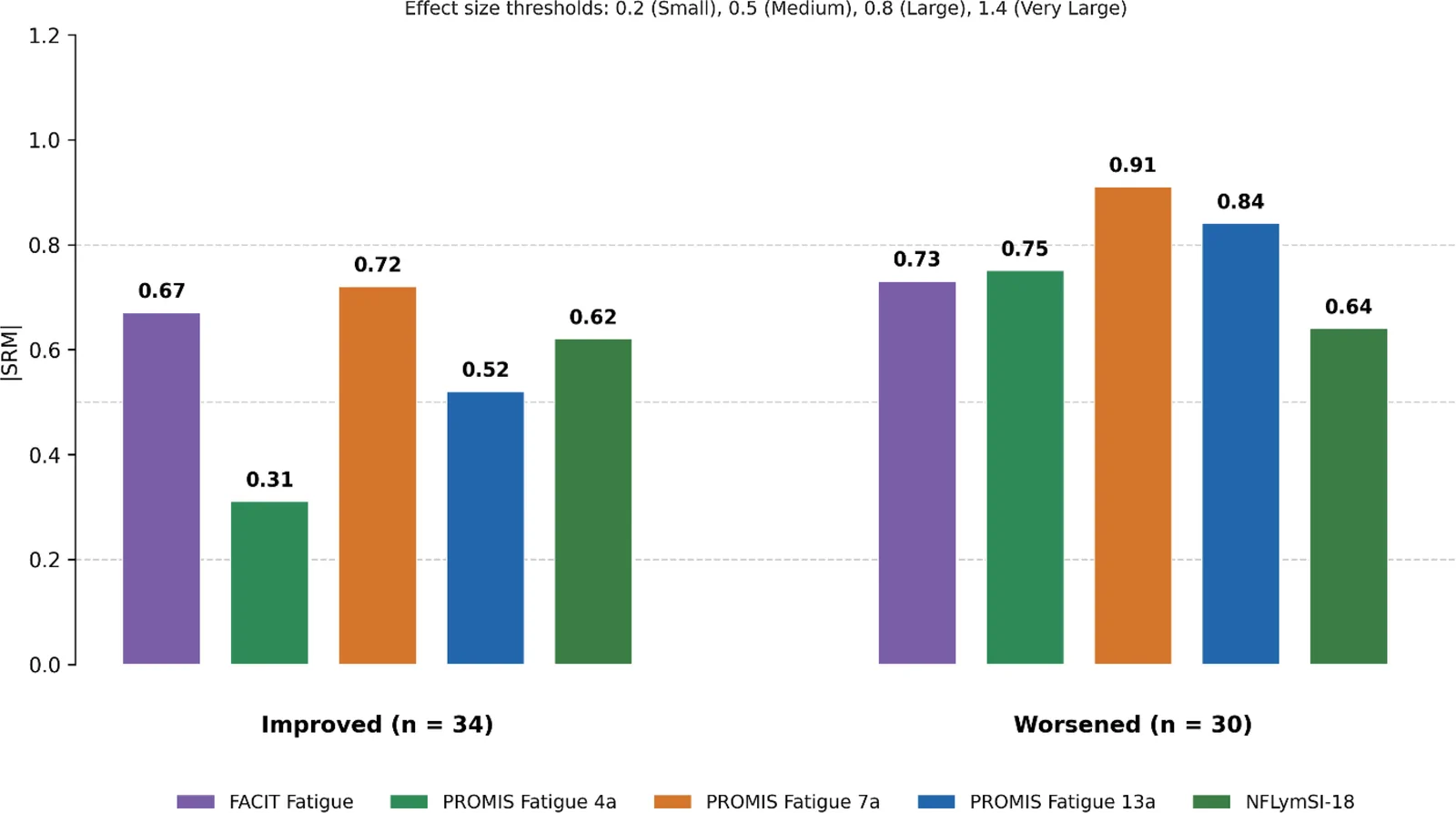
Standardized response mean effect sizes by patient global rating of change (PGRC): Improved vs. Worsened (absolute values). Patient Global Rating of Change (PGRC) at 3 month follow-up measures perceived change in fatigue with a question: “Thinking about your experience of fatigue, since the last time you filled out a questionnaire for this project, your fatigue is…”

**Fig. 1b:**
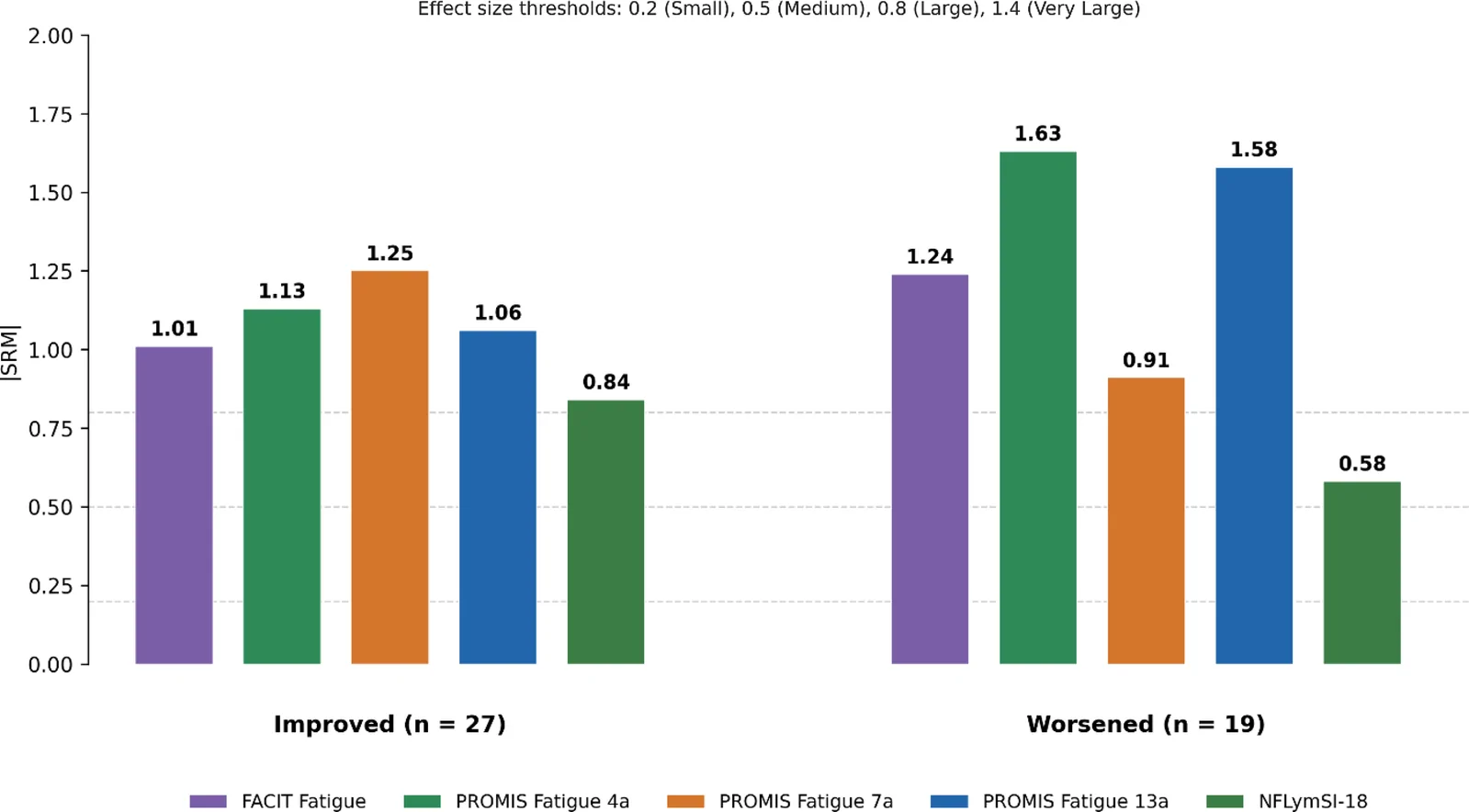
Standardized response means by patient global rating of severity (PGRS1): Improved vs. Worsened (absolute values). Patient Global Rating of Severity (PGRS1) at 3-month follow-up: “Thinking about your experience of fatigue, how severe has your fatigue been in the past 7 days?”

**Fig. 1c:**
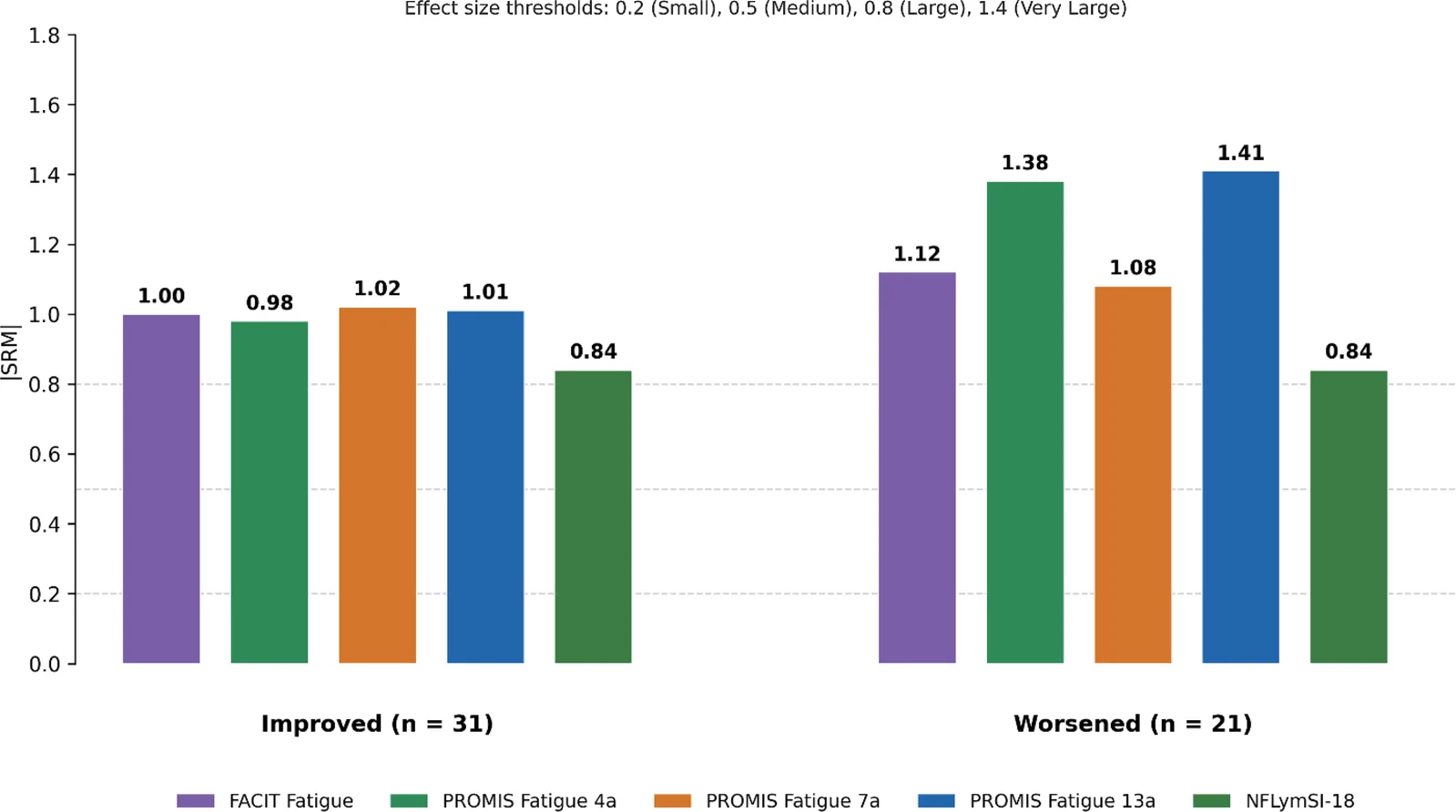
Standardized response mean effect sizes by patient global rating of fatigue interference (PGRS2): Improved vs. Worsened (absolute values). Patient Global Rating of Severity for fatigue interference (PGRS2) measures the impact of fatigue on daily functioning: “*Thinking about your experience of fatigue*,* how much has fatigue interfered with your life in the past 7 days?”*

**Fig. 1d:**
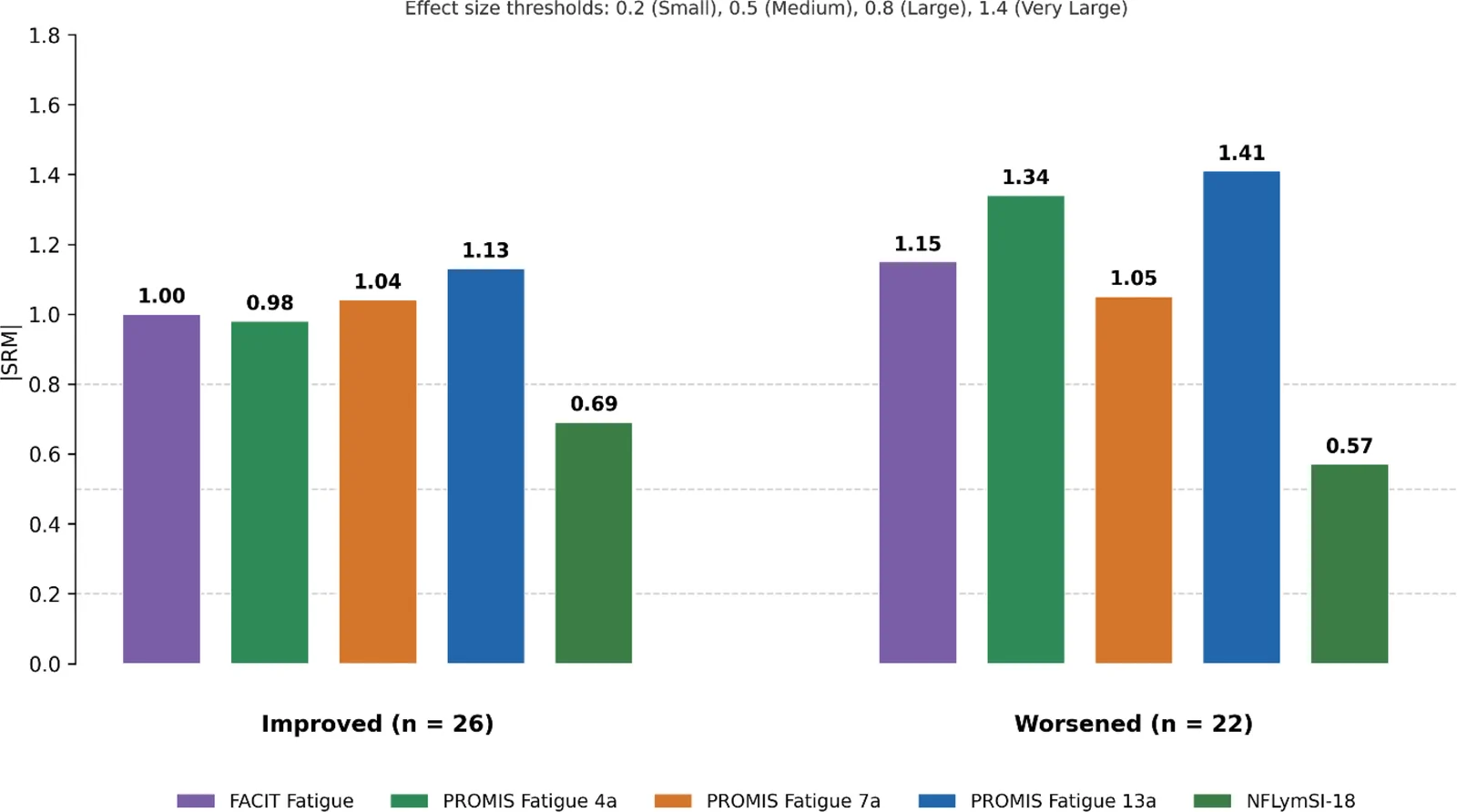
Standardized response mean effect sizes by the patient-reported outcomes version of the common terminology criteria for adverse events (PRO-CTCAE) fatigue severity: Improved vs. Worsened (absolute values). In the last 7 days, what was the severity of your fatigue, tiredness or lack of energy at its worst?

**Fig. 1e:**
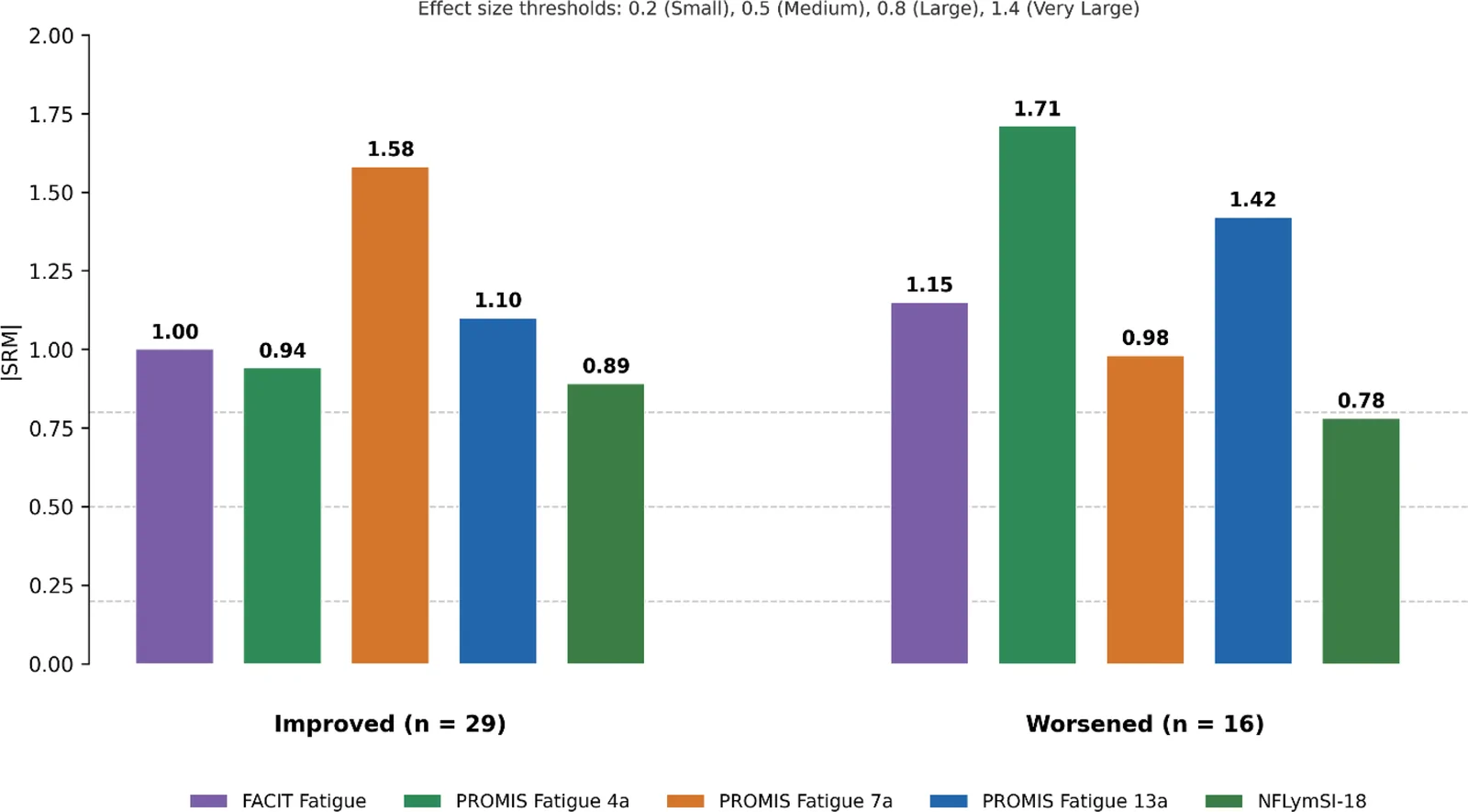
Standardized response mean effect sizes by the patient-reported outcomes version of the common terminology criteria for adverse events (PRO-CTCAE) Fatigue Interference: Improved vs. Worsened (absolute values). CTCAE Interference: Measures how much fatigue interferes with activities of daily living In the last 7 days, how much did fatigue, tiredness or lack of energy interfere with your usual or daily activities?

#### Patient global rating of severity (PGRS1) and interference (PGRS2)

As shown in Fig. 1b, SRM values were generally higher with the PGRS1 anchor compared to the PGRC anchor. Most measures exceeded the threshold for large effect size (SRM > 0.8) in both improvement and worsening directions. The PROMIS Fatigue 7a demonstrated the largest SRM for improvement (1.25; mean change − 6.3), followed by PROMIS Fatigue 4a (1.13; mean change − 8.1), PROMIS Fatigue 13a (1.06; mean change − 6.1), FACIT-Fatigue (1.01; mean change + 7.6), and NFLymSI-18 (0.84; mean change + 6.8). For worsening, PROMIS Fatigue 4a showed the largest SRM (1.63; mean change + 11.5), followed by PROMIS Fatigue 13a (1.58; mean change + 9.2), FACIT-Fatigue (1.24; mean change − 10.9), PROMIS Fatigue 7a (0.91; mean change + 7.6), and NFLymSI-18 (0.58; mean change − 5.9).

For the PGRS2 anchor, SRM patterns (Fig. 1c) were similar to those observed with the PGRS1 anchor. PROMIS Fatigue 7a demonstrated the largest SRM for improvement (1.02; mean change − 5.4), followed by PROMIS Fatigue 13a (1.01; mean change − 5.7), FACIT-Fatigue (1.00; mean change + 7.0), PROMIS Fatigue 4a (0.98; mean change − 7.1), and NFLymSI-18 (0.84; mean change + 6.1). For worsening, PROMIS Fatigue 13a showed the largest SRM (1.41; mean change + 8.3), followed by PROMIS Fatigue 4a (1.38; mean change + 10.3), FACIT-Fatigue (1.12; mean change − 9.7), PROMIS Fatigue 7a (1.08; mean change + 7.9), and NFLymSI-18 (0.84; mean change − 6.4).

#### PRO-CTCAE fatigue anchors

Using the PRO-CTCAE fatigue severity item as an anchor, SRM patterns (Fig. 1d) showed PROMIS Fatigue 13a to be most responsive to improvement (SRM = 1.13; mean change − 6.5), followed by PROMIS Fatigue 7a (1.04; mean change − 5.3), FACIT-Fatigue (1.00; mean change + 7.1), PROMIS Fatigue 4a (0.98; mean change − 7.6), and NFLymSI-18 (0.69; mean change + 5.3). For worsening, PROMIS Fatigue 13a was again most responsive (SRM = 1.41; mean change + 8.3), followed by PROMIS Fatigue 4a (1.34; mean change + 9.9), FACIT-Fatigue (1.15; mean change − 9.8), PROMIS Fatigue 7a (1.05; mean change + 7.8), and NFLymSI-18 (0.57; mean change − 5.3).

For the PRO-CTCAE fatigue interference item (Fig. 1e), PROMIS Fatigue 7a showed the largest SRM for improvement (1.58; mean change − 7.5), followed by PROMIS Fatigue 13a (1.10; mean change − 6.0), FACIT-Fatigue (1.00; mean change + 7.3), PROMIS Fatigue 4a (0.94; mean change − 7.0), and NFLymSI-18 (0.89; mean change + 5.4). For worsening, PROMIS Fatigue 4a showed the largest SRM (1.71; mean change + 12.3), followed by PROMIS Fatigue 13a (1.42; mean change + 9.3), FACIT-Fatigue (1.15; mean change − 11.3), PROMIS Fatigue 7a (0.98; mean change + 7.9), and NFLymSI-18 (0.78; mean change − 7.8).

### Statistically significant individual-level change thresholds

**Fig. 2:**
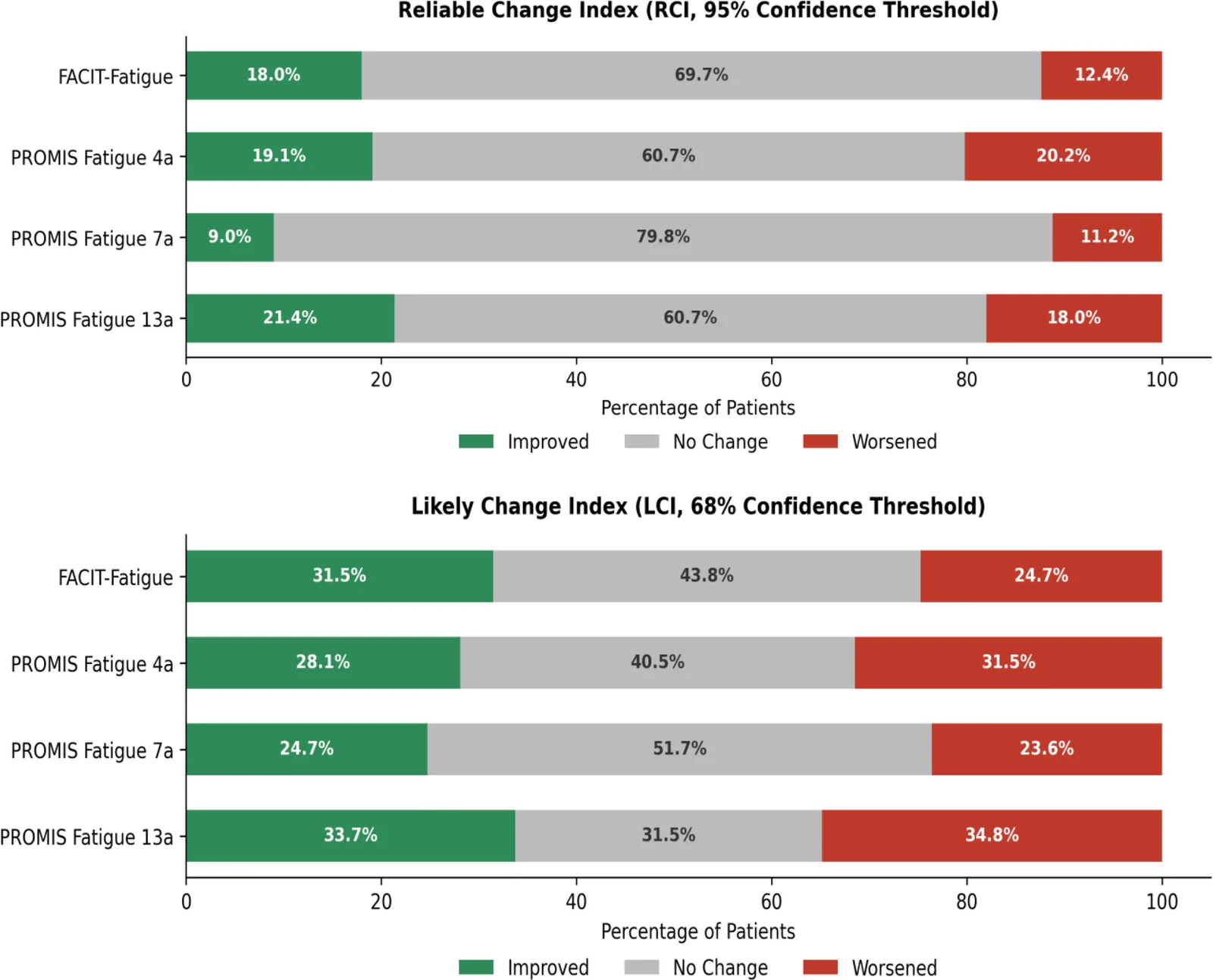
Proportion of patients with reliable individual-level fatigue change by RCI (95% CI) and LCI (68% CI) thresholds. This figure displays the percentage of patients classified as “Improved,” “No Change,” or “Worsened” across six fatigue measures (FACIT-Fatigue, PROMIS Fatigue 4a, 7a, 13a, and NFLymSI-18), using both the Reliable Change Index (RCI, 95% confidence threshold) and the Likely Change Index (LCI, 68% confidence threshold)

Using the more conservative 95% confidence threshold for RCI (*p* < 0.05), statistically significant improvement was identified in 18.0% of patients using FACIT-Fatigue, compared to 21.4%, 9.0%, and 19.1% using PROMIS Fatigue 13a, 7a, and 4a, respectively. For worsening with the 95% RCI, PROMIS Fatigue 4a identified the highest percentage (20.2%), followed by PROMIS Fatigue 13a (18.0%), FACIT-Fatigue (12.4%), and PROMIS Fatigue 7a (11.2%) (Fig. 2).

With the less stringent 68% confidence threshold for LCI (*p* < 0.32), PROMIS Fatigue 13a classified the highest percentage of patients as improved (33.7%), followed by FACIT-Fatigue (31.5%), PROMIS Fatigue 4a (28.1%), and PROMIS Fatigue 7a (24.7%). For worsening, PROMIS Fatigue 13a again identified the highest percentage (34.8%), followed by PROMIS Fatigue 4a (31.5%), FACIT-Fatigue (24.7%), and PROMIS Fatigue 7a (23.6%). The roughly symmetric proportions of patients classified as improved and worsened are consistent with the heterogeneous clinical trajectories in this observational cohort, where patients were at varying points in their treatment course.

## Discussion

Our study evaluating responsiveness of the FACIT-Fatigue, NFLymSI-18 and multiple PROMIS Fatigue measures (4a, 7a and 13a) in patients with lymphoma reveals important patterns to guide measure selection in clinical trials. Both measurement systems demonstrated good responsiveness to change, though responsiveness patterns varied considerably by anchor, direction of change, and specific measure. No single measure consistently showed superior responsiveness across all conditions. For the PGRC anchor, PROMIS Fatigue 7a showed the best responsiveness for both improvement and worsening. However, for other anchors, different measures emerged as most responsive - for example, PROMIS Fatigue 4a and 13a showed the largest SRMs for worsening with PGRS1, while PROMIS Fatigue 7a showed largest responsiveness for improvement with PRO-CTCAE interference. Our findings on anchor selection also have methodological implications. Measures showed stronger responsiveness with PGRS1 and PGRS2 compared to PGRC. This aligns with research suggesting current state comparisons may be more reliable than retrospective assessments, which are subject to recall bias and influence from current status [26]. 

These results have direct implications for clinical trial design in lymphoma. The variable responsiveness patterns suggest that measure selection should be tailored to the specific trial context. Notably, the differential responsiveness between FACIT-Fatigue and PROMIS Fatigue 13a -- despite containing identical items -- highlights the critical impact of scoring methodology. The FACIT-Fatigue uses traditional sum based scoring while PROMIS employs Item Response Theory (IRT)-based T-scores. IRT-based T-scores have a non-linear relationship with raw sum scores [7]. In the middle range of the T-score distribution where most patients score, large raw score changes translate to smaller T-score changes. Conversely, at the extremes of the distribution, smaller raw score changes yield larger T-score changes. This may explain why PROMIS 13a showed larger SRMs for worsening (where scores move toward the extreme) compared to improvement in several anchors. For comprehensive assessment, researchers might consider including multiple measures to optimize detection of change, resources permitting. Formal derivation of meaningful score differences through triangulation of anchor-based and distribution-based approaches for these measures in lymphoma is warranted in future research.

Several limitations should be noted. Our sample size, particularly when stratified by specific anchors, may limit precision of our estimates. Additionally, our method for defining ‘improvement’ and ‘worsening’ using single-level changes on anchor measures may have influenced responsiveness estimates. This approach treats all one-level changes as equivalent, potentially combining minimal changes with more substantial improvements or deteriorations, which could affect the interpretation of responsiveness patterns. Second, the 3-month follow-up may not capture optimal time points for maximal change given variable treatment trajectories. Finally, these results may not fully generalize to all lymphoma subtypes. Additionally, Groups A and B differed in their treatment status at baseline (prior to starting next line of therapy vs. on active treatment), which may have influenced fatigue trajectories and responsiveness estimates. The relatively small subgroup sample sizes when stratified by anchor category (*n* = 16 to 44) may limit the precision of SRM estimates. Our sample was predominantly White (87.6%) and non-Hispanic/Latino (83.1%), limiting generalizability; future research should evaluate the responsiveness of these instruments in more racially and ethnically diverse populations to determine whether cultural factors influence reliability and sensitivity of these measures. Future research should explore whether these results persist in larger samples, across different lymphoma classifications, and with varied follow-up periods.

## Conclusion

Overall, the findings demonstrate that the FACIT-Fatigue, NFLymSI-18 and PROMIS Fatigue measures (4a, 7a, 13a) all show good responsiveness to change in patients with lymphoma, with no single measure demonstrating consistent superiority across all anchors and directions of change. The complex patterns observed - including the impact of scoring methodology (sum scores versus IRT-based T-scores) on responsiveness - highlight the importance of careful measure selection based on the specific research context, anticipated direction of change, and chosen anchors. For studies where both improvement and deterioration are important to detect, using complementary measurement systems may provide the most comprehensive assessment of fatigue changes in patients with lymphoma.

## Supplementary Information

Below is the link to the electronic supplementary material.

Supplementary Material 1

Supplementary Material 2

## Data Availability

The datasets generated and/or analyzed during the current study are available from the corresponding author on reasonable request.

## Abbreviations

ECOG: Eastern Cooperative Oncology Group
FACIT-Fatigue: Functional Assessment of Chronic Illness Therapy–Fatigue
IRT: Item Response Theory
LCI: Likely Change Index
NFLymSI-18: National Comprehensive Cancer Network–Functional Assessment of Cancer Therapy Lymphoma Symptom Index (18-item version)
PGRC: Patient Global Rating of Change
PGRS1: Patient Global Rating of Severity
PGRS2: Patient Global Rating of Interference
PRO: Patient-Reported Outcome
PRO-CTCAE: Patient-Reported Outcomes version of the Common Terminology Criteria for Adverse Events
PROMIS: Patient-Reported Outcomes Measurement Information System
RCT: Randomized Controlled Trial
RCI: Reliable Change Index
SD: Standard Deviation
SRM: Standardized Response Mean
