# Supplementary material for: Measuring group‑ and individual‑level change of FACIT- fatigue, NFLymSI-18 and PROMIS fatigue measures (4a, 7a and 13a) in lymphoma
Source: J Patient Rep Outcomes. 2026 Jul 19;10:191. doi: 10.1186/s41687-026-01154-6 (PMC13672741; doi:10.1186/s41687-026-01154-6)
Supplement: Supplementary file 1 [file 41687_2026_1154_MOESM1_ESM.docx]

**Supplementary Files**

**Table S1. Spearman Correlation Coefficients Between Fatigue Measure Change Scores and Anchor Variables**

| **Fatigue Measure** | **PGRC** | **PGRS1** | **PGRS2** | **CTCAE-Severity** | **CTCAE-Interference** |
| --- | --- | --- | --- | --- | --- |
| FACIT-Fatigue | 0.54 | –0.76 | –0.76 | –0.73 | –0.73 |
| PROMIS-Fatigue4a | –0.43 | 0.75 | 0.74 | 0.72 | 0.75 |
| PROMIS-Fatigue7a | –0.60 | 0.67 | 0.71 | 0.63 | 0.77 |
| PROMIS-Fatigue13a | –0.52 | 0.73 | 0.73 | 0.73 | 0.72 |
| FLYMSI-18 Total | 0.58 | -0.58 | -0.59 | -0.52 | -0.57 |

*Note: PGRC = Patient Global Rating of Change; PGRS1 = Patient Global Rating of Severity; PGRS2 = Patient Global Rating of Interference; CTCAE-Severity = Common Terminology Criteria for Adverse Events Fatigue Severity item; CTCAE-Interference = Common Terminology Criteria for Adverse Events Fatigue Interference item. Values shown are Spearman correlation coefficients. All correlations are statistically significant at p<0.05.*
