# Supplementary material for: Measuring group‑ and individual‑level change of FACIT- fatigue, NFLymSI-18 and PROMIS fatigue measures (4a, 7a and 13a) in lymphoma
Source: J Patient Rep Outcomes. 2026 Jul 19;10:191. doi: 10.1186/s41687-026-01154-6 (PMC13672741; doi:10.1186/s41687-026-01154-6)
Supplement: Supplementary file 2 [file 41687_2026_1154_MOESM2_ESM.docx]

**Table S2.** Standardized Response Mean (SRM) Values by Detailed Patient Global Rating of Change (PGRC) Response Categories

| **PGRC Category** | **n** | **FACIT- Fatigue** | **PROMIS Fatigue 4a** | **PROMIS Fatigue 7a** | **PROMIS Fatigue 13a** | **NFLymSI-18** |
| --- | --- | --- | --- | --- | --- | --- |
| Minimally Improved (PGRC = +1) | 7 | 0.76 | 0.22 | 0.25 | 0.56 | 0.78 |
| Much Improved (PGRC = +2/+3) | 27 | 1.04 | 0.41 | 0.85 | 0.43 | 0.58 |
| No Change (PGRC = 0) | 25 | 0.91 | 0.23 | 0.47 | 0.06 | 0.32 |
| Minimally Worsened (PGRC = -1) | 14 | 0.55 | 0.86 | 1.06 | 0.97 | 0.35 |
| Much Worsened (PGRC = -2/-3) | 14 | 0.81 | 0.87 | 0.88 | 0.74 | 1.12 |
| **Pooled Improved (PGRC ≥ +1)** | **34** | **0.67** | **0.31** | **0.72** | **0.52** | **0.62** |
| **Pooled Worsened (PGRC ≤ -1)** | **30** | **0.73** | **0.75** | **0.91** | **0.84** | **0.64** |

*Note: All values are absolute SRM values. For FACIT-Fatigue and NFLymSI-18, positive change scores indicate improvement. For PROMIS Fatigue measures, negative change scores indicate improvement. Pooled values (bottom rows, bold) correspond to the SRMs reported in the main manuscript (Figure 1a). The dose-response pattern (larger SRMs in the "Much" changed categories compared to "Minimally" changed categories) confirms that pooling response levels did not artificially inflate responsiveness estimates. Small cell sizes in individual response categories (e.g., n = 7 for Minimally Improved) motivated the decision to pool categories for the primary analysis.*

*PGRC response options: +3 = Very much better (n = 15); +2 = Moderately better (n = 12); +1 = A little better (n = 7); 0 = About the same (n = 25); -1 = A little worse (n = 14); -2 = Moderately worse (n = 12); -3 = Very much worse (n = 2).*
